# Characterizing the Evolutionary Path(s) to Early *Homo*

**DOI:** 10.1371/journal.pone.0114307

**Published:** 2014-12-03

**Authors:** Lauren Schroeder, Charles C. Roseman, James M. Cheverud, Rebecca R. Ackermann

**Affiliations:** 1 Department of Archaeology, University of Cape Town, Rondebosch, 7701, South Africa; 2 Department of Anthropology, University of Illinois, Urbana-Champaign, Illinois, United States of America; 3 Department of Biology, Loyola University, Chicago, Illinois, United States of America; Duke University School of Medicine, United States of America

## Abstract

Numerous studies suggest that the transition from *Australopithecus* to *Homo* was characterized by evolutionary innovation, resulting in the emergence and coexistence of a diversity of forms. However, the evolutionary processes necessary to drive such a transition have not been examined. Here, we apply statistical tests developed from quantitative evolutionary theory to assess whether morphological differences among late australopith and early *Homo* species in Africa have been shaped by natural selection. Where selection is demonstrated, we identify aspects of morphology that were most likely under selective pressure, and determine the nature (type, rate) of that selection. Results demonstrate that selection must be invoked to explain an *Au. africanus*—*Au. sediba*—*Homo* transition, while transitions from late australopiths to various early *Homo* species that exclude *Au. sediba* can be achieved through drift alone. Rate tests indicate that selection is largely directional, acting to rapidly differentiate these taxa. Reconstructions of patterns of directional selection needed to drive the *Au. africanus*—*Au. sediba*—*Homo* transition suggest that selection would have affected all regions of the skull. These results may indicate that an evolutionary path to *Homo* without *Au. sediba* is the simpler path and/or provide evidence that this pathway involved more reliance on cultural adaptations to cope with environmental change.

## Introduction

Recent research proposes that habitat instability and fragmentation acted as important environmental forces driving the evolution and diversification of early *Homo* [1]. In the authors' scenario, the biological and behavioural adaptations associated with early *Homo* evolved not as a package but rather in an experimental manner over a considerable time frame. This suggests that the transition from *Australopithecus* to early *Homo* was not simple, and resulted in multiple lineages within early *Homo* [1]. The substantial morphological variation in the *H. erectus* samples from Dmanisi, Georgia (∼1.8 Ma), and the mosaic features in the contemporaneous African species *Au. sediba* (∼1.98 Ma), add further weight to the idea that the emergence of our genus was characterized by evolutionary experimentation/innovation resulting in diverse morphology. This increasing recognition of the evolution of early *Homo* as multi-branched, or bushy [1]–[5], in turn implies that there are multiple ways to produce early *Homo*. However, details of the evolutionary processes underlying this transition and the production of morphological variation are poorly understood. Most explanations for hominin diversity in the early Pleistocene, and especially hypotheses about the emergence of the genus *Homo*, assume that major evolutionary changes are adaptive given their co-occurrence with substantial environmental change in Africa at this time [1], [6]–[11]. However, quantitative analyses of diversity within *Homo* – albeit limited – suggest that genetic drift may play an important role in producing evolutionary change [12], [13]. Each of these scenarios has important implications relevant to the longstanding debate about the relative importance of neutral versus adaptive evolution in shaping organismal form [14]–[18]. In this context, differentiating among potential drivers of evolution can provide insight into the means by which evolution acted to produce our genus. Determining the role of selection in shaping morphology might also allow for recognition of the relative importance of biological versus behavioral adaptation in our lineage.

Here, we characterize the evolutionary processes necessary to transition from australopiths to early *Homo*. We test hypotheses of evolutionary diversification in the early Pleistocene human fossil record to determine whether cranial diversity (face, neurocranium and mandible) can be explained solely by genetic drift, or whether non-random forces (i.e. selection) must be invoked. In cases where selection is apparent, we identify aspects of morphology that were most likely under selective pressure, and determine the nature (type, rate) of that selection. We focus on the transition from australopiths to *Homo*, determining the evolutionary forces that would have been necessary to evolve *Homo* from our generalized australopith model, *Au. africanus*. We do not make any assumptions about which morphotype of early *Homo* (e.g. *Homo habilis* versus *Homo rudolfensis*) is the correct transition but rather look at all possible paths. We also consider an alternative path that includes *Au. sediba*, given the possibility that *Au. sediba* is a transitional species between australopiths and our genus [19], [20], determining the evolutionary forces necessary to evolve *Au. sediba* from *Au. africanus*, and various lineages of *Homo* from *Au. sediba*.

## Materials and Methods

### Samples and data acquisition

The fossil specimens used in this study are as follows: *Australopithecus africanus* (MLD 2, MLD 40, Sts 5, Sts 7, Sts 36, Sts 52, Sts 71, Stw 13, Stw 327, Stw 505, Stw 513), *Au. sediba* (MH1, MH2), *Homo habilis* (KMN-ER 820, KNM-ER 1501, KNM-ER 1805, KNM-ER 1813, OH 13, OH 24, OH 37), South African early *Homo* (SK 15, SK 45, SK 847, Stw 53), *H. rudolfensis* (KMN-ER 1470, KNM-ER 1482, KNM-ER 1801, KNM-ER 1802), *H. erectus* (KNM-BK 67, KNM-BK 8518, KNM-ER 730, KNM-ER 992, KNM-ER 3733, KNM-ER 3734, KNM-ER 3883, KNM-ER 42700, KNM-WT 15000, OH 22). Specimen choice was dependent on the availability of certain landmarks. Some specimens and/or variables were omitted from analyses due to the lack of visible sutural landmarks, preservation or distortion. All necessary permits were obtained for the described study, which complied with all relevant regulations. A detailed description of fossil and extant samples utilized in each analysis, as well as repository and permit information, can be found in Text S1.

All fossil and extant material were scanned using a NextEngine Desktop 3D Laser scanner, and digital surfaces were modeled. Three-dimensional landmarks representing homologous structures across species were plotted directly on the reconstructed surfaces, and Euclidean distances were derived from these 3D coordinates (Text S1 and Table 1). The choice of variables was dictated by the shared preservation of the fossil specimens. The number and distribution of landmarks are sufficient for identifying differences between the extant species. Separate analyses focus on different regions of the skull to maximize the specimens available for study, since most hominin fossils are fragmentary. Therefore each analysis may involve different representatives of the various taxa. All analyses are done with raw data to evaluate differentiation in both size and shape.

**Table 1:** Standardized landmarks recorded from crania and mandibles.

**Cranial Landmarks**		
**Landmark abbreviation**	**Landmark** a	**Landmark definition**
ANS	Anterior nasal spine	The most anterior point on the maxilla
PRO	Prosthion	The most anterior point in the midline of the maxillary alveolar process
IOF	Infraorbital foramen	The most inferior lateral point on the border of the infraorbital foramen
ALR	Alare	The most lateral point on the nasal aperture
OR	Orbitale	The most inferior point on the midpoint of the lower edge of the orbit
SON	Supraorbital notches	The most lateral point on the supraorbital notch
DAC	Dacryon	The point of intersection of the frontolacrimal and lacrimomaxillary sutures
NA	Nasion	The point at the intersection of the nasofrontal suture and the midsagittal plane
FMT	Frontomalare temporale	The most lateral point on the frontozygomatic suture
ZMI	Zygomaxillare inferior	The most inferior point on the zygomaxillary suture
AP	Anterior pterion	The most anterior point on the sphenoparietal suture/the intersection of the parietal, sphenoid and frontal bones
POR	Porion	The most superior point on the margin of the external auditory meatus
MT	Maxillary tuberosity	The most distal point on the maxillary alveolar process
PN	Parietal notch	The indentation or angle between the squamous and petrous parts of the temporal bone, taken on the superior border of the squama temporalis
BR	Bregma	The midline junction of the coronal and sagittal sutures
ALV	Alveolare	The most anterior point on the alveolus of the M1
MFL	Lateral mandibular fossa	The most lateral point on the mandibular fossa
**Mandibular Landmarks**		
MMN	Mid-mandibular notch	The most inferior aspect on the mid-mandibular notch
AJUNC	Inferior anterior ramus	The junction of the anterior border of the ramus and alveolus
GON	Gonian	The junction of the ramus and inferior border of body
MEN	Mental foramen	The most anteromedial edge of the mental foramen
INFR	Infradentale	The most central point on the mandibular alveolus
MFO	Mandibular foramen	The most posteroinferior aspect on the mandibular foramen
MSPIN	Superior mental spine	The most superior aspect on the mental spine
M2D	Distal m2	The most distal point in the midline of m2
M2M	Mesial m2	The most mesial point in the midline of m2
M1M	Mesial m1	The most mesial point in the midline of m1
ALV	Alveolar border of body	The most superior point on the alveolus directly above the mental foramen
IBB	Inferior border of body	The most inferior point on the mandibular corpus directly below the mental foramen

a Landmarks and measurements are derived from laser surface scans taken by LS. Landmarks are adapted from [48]–[56].

Inter-landmark distances are drawn from these landmarks for each of the 10 analyses.

### Testing hypotheses of genetic drift

The methodological approach taken here derives from the quantitative evolutionary theory of Lande [21]–[23]. Following Ackermann and Cheverud [12], [24], the hypothesis of proportionality of between-group phenotypic variation and within-group phenotypic variation 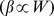 is tested. Proportionality indicates that diversification of the taxa can be explained by random genetic drift, while lack of proportionality indicates that non-random evolutionary processes, such as directional selection, are likely to be at work. Phenotypic within-population variance/covariance (V/CV) matrices derived from humans (*Homo sapiens*) and chimpanzees (*Pan troglodytes*) were used as models for hominin within-population variability (see [12]), and were then simplified to their principal components (PCs). We used both human and chimp matrices to conservatively account for the possible effects of small differences in within population covariance structure across fossil and extant taxa, and because the fossils considered here are both ape-like (i.e. australopiths) and human-like (i.e. *Homo*). Means of available individuals for each taxon (between two and seven individuals), and occasionally values for an individual specimen, are taken to represent population means for the fossils. PC scores were calculated for each fossil population by multiplying trait means by the standardized within-population loadings; between-population variance for each PC was then calculated as the variance among these population mean PC scores. Between- and within-population variances (eigenvalues) were then logged. On a logarithmic scale, we expect a regression slope of 1.0 for the regression of between- on within-population variance if differentiation has been produced by random genetic drift; non-significant or no deviation means that we have failed to reject drift. The inability to reject drift does not eliminate the possibility that selection occurred, but rather indicates that any effect of selection cannot be distinguished from chance changes caused by drift. A significant deviation from a slope of 1.0 indicates a pattern that is likely to have been caused by nonrandom (e.g. selective) processes. By design, this test makes it difficult to reject drift when sample sizes are small and few taxa are being compared. Therefore, it is likely that any indication of significant departure from proportionality under either extant model will indicate selection. All calculations were done in R version 3.0.1.

### Rates of evolution

To investigate the nature of the potential selection acting on these populations, neutral hypotheses of rates of evolution were tested using Lande's generalized genetic distance [22] for each hypothesized ancestor to descendent transition, focusing on transitions where selection was detected (i.e. in the *Au. africanus* – *Au. sediba* – *Homo* transition). This distance statistic allows a two tailed test in which small values reflect the action of stabilizing selection (divergence is too slow for drift) and large values indicate directional selection (divergence is too fast) [22]. Four different estimates were obtained for each transition using either the chimpanzee or human covariance matrix 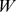
taken as a whole, or multiplied by 0.4 to reflect the fact that morphological characteristics are imperfectly heritable. The 0.4 value was chosen because it is close to the modal value for heritability in cranial characteristics in a primate model [25]. The full estimate of P will give a minimum rate of evolution while the imperfectly heritable case will give a higher expected rate [22]. A generation length of 25 years [26] and an effective population size of 21,000 individuals [27] were chosen to resemble a chimpanzee population. Asymptotically, the generalized genetic distance is expected to be chi-square distributed under drift with the number of degrees of freedom equal to the number of traits. The small sample sizes of the fossil groups used here, however, will cause the estimated distances to be biased toward values larger than the true value. Test distributions that included this sampling bias were constructed using simulations that had both an evolutionary component that tracked drift in the vector of population means and a sampling component that took into account the number of individuals from each fossil group in a comparison. This method differs from the slope test for genetic drift in that it takes advantage of both direction of divergence and overall magnitude of divergence whereas the other is concerned with direction in the form of proportionality.

### Reconstructing selection

When a null hypothesis of drift is rejected, we reconstruct the selection necessary to produce the differentiation in observed population means, using the following relationship: 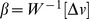 where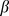 is the differential selection gradient summed over the generations [28], 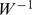 is the inverse of the pooled within-species phenotypic V/CV matrix, and 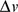 is the vector of the differences in variable means between the fossil species being compared [24]. Again, the extant phenotypic V/CV matrices are used as models for fossil variation. We know that V/CV structures are not strictly constant through time, and moreover there may be differences between fossil CV structure and that of the extant models, and therefore these selection vectors should be interpreted cautiously, as guides to the general pattern (but perhaps not the precise magnitude) of selection.

For all the analyses we make the assumption that *Au. africanus* represents a likely ancestor (or is similar to such an ancestor) for early *Homo*, and/or for *Au. sediba*. Because of the inclusion of *Au. sediba* in this analysis, using *Au. africanus* as the ancestral form is likely to be more credible than using another australopith species (e.g. *Au. afarensis*); whether the substitution of *Au. afarensis* alters the results remains to be tested. The analyses are structured hierarchically to focus on the relationships between temporally successive hominins in three ways. First, we look across all of the australopith and *Homo* taxa to test for neutral versus adaptive divergence. Second, we focus in on the relationship between *Au. africanus* and *Homo*, running individual tests between this model australopith and each of the sampled early *Homo* species (including a sample from South Africa whose specific affiliation is uncertain). Third, we examine the relationship between *Au. sediba* and *Au. africanus*, and between *Au. sediba* and *Homo*, again running individual tests between *Au. sediba* and each of the early *Homo* species. This methodological approach has been shown to work at different levels in a phylogeny, and therefore the analysis can be considered robust whether or not the taxa represent different species, or are time-successive species [12], [24], [29], [30]. Importantly, *Au. sediba* is represented by only two individuals, one of which is a nearly-mature juvenile at the stage of pre-M3 eruption; the possible effect this might have on the analyses is discussed when appropriate.

## Results

In total, ten sets of analyses were performed on different subsets of variables from the face, neurocranium, and mandible. The regressions of logged eigenvalues of between-group variation against logged eigenvalues of within-group variation are shown in Table 2. These analyses indicate that in most instances the phenotypic diversity seen across these taxa is consistent with random evolutionary processes – i.e. genetic drift. However, there are five comparisons which generate statistically significant p-values less than or equal to 0.05 (Figure S1). All of these occur when considering the transition from *Au. africanus* to *Homo* that includes *Au. sediba* as in intermediate. In the first two sets of analyses, which focus on variables in the face, drift cannot be rejected as the cause of diversification. This is true whether all of the hominins are considered together, whether analyses are performed on subsets of these hominins, and regardless of the extant model (human or chimpanzee) used to simulate within-taxon variation. This suggests that covariance among characteristics is probably structuring evolutionary trajectories to a large extent, and may mean that random processes played a role in the diversification of these regions of the face across this morphologically diverse group of hominins. It does not mean that selection did not play a role in other facial regions, and indeed the third and fourth analyses that focus on the face indicate some effect of selection. For the third analysis, which focuses on variables in the midface, it was not possible to reject drift for most comparisons, however drift is rejected in the comparison between *Au. sediba* and a South African member of the genus *Homo* (SK 847) under a human model of within-population variation. In the fourth analysis, which contains variables in the maxillary and zygomatic region, drift is rejected in the comparison of *Au. africanus* to *Au. sediba* under both extant models of variation, and possibly in the comparison of *Au. sediba* with *H. erectus*. In the fifth cranial analysis, drift is rejected in the comparison among all groups, with significant and borderline significant values depending on the extant model, and between *Au. sediba* and *H. erectus* (again, significant or borderline depending on whether the model is chimp or human, respectively). Negative or positive deviations of regression lines from a slope of 1.0 indicate more or less between-group variation, respectively, than expected under a model of drift. For all of these analyses of the cranium where drift was rejected, the slope is greater than 1.0, indicating that the first few PCs show greater than expected between-fossil variation, while the minor components display less than expected. It is, however, important to note that the *Au. sediba* specimen used in these cranial analyses is a juvenile (MH1); presumably we would expect more difference between this juvenile and the other taxa (all adults) than we would see in an adult. However, in an analysis of facial growth in macaques, Cheverud and Richtsmeier [31] found that basic shape changes are minimal between adults and juveniles at the age of MH1. Similarly, Ponce de León and Zollikofer [32] found that species characteristic differences between *H. neanderthalensis* and *H. sapiens* in facial and mandibular morphology are evident very early during growth, well before the developmental stage represented by MH1. We therefore interpret the effect of the juvenile status of the specimen as negligible.

**Table 2:** Results of between-group variance regressed on within-group variance as a test for genetic drift.

Analysis	Comparison	Extant model	Consistent with drift?	Slope	*R^2^*	*p*-value
**Cranial Analysis 1**	All groups	Human	Yes	0.95	0.84	0.77
**Face**		Chimp	Yes	0.65	0.47	0.27
	*Au. africanus* – SA early *Homo*	Human	Yes	0.87	0.39	0.79
		Chimp	Yes	1.01	0.30	0.99
	*Au. africanus* – *H. habilis*	Human	Yes	0.79	0.70	0.36
		Chimp	Yes	0.69	0.24	0.56
	*Au. africanus* – *H. rudolfensis*	Human	Yes	1.13	0.54	0.77
		Chimp	Yes	0.55	0.07	0.61
	*Au. africanus* – *Au. sediba*	Human	Yes	0.34	0.07	0.23
		Chimp	Yes	0.92	0.45	0.85
	*Au. sediba* – SA early *Homo*	Human	Yes	0.73	0.50	0.39
		Chimp	Yes	1.16	0.42	0.78
	*Au. sediba* – *H. habilis*	Human	Yes	0.61	0.22	0.44
		Chimp	Yes	0.31	0.04	0.34
	*Au. sediba* – *H. rudolfensis*	Human	Yes	1.14	0.45	0.79
		Chimp	Yes	0.57	0.21	0.37
**Cranial Analysis 2**	All groups	Human	Yes	0.88	0.72	0.51
**Face**		Chimp	Yes	0.99	0.97	0.82
	*Au. africanus* – *H. habilis*	Human	Yes	1.18	0.74	0.44
		Chimp	Yes	1.19	0.54	0.59
	*Au. africanus* – *H. erectus*	Human	Yes	0.61	0.21	0.32
		Chimp	Maybe	0.51	0.32	**0.06**
	*Au. africanus – Au. sediba*	Human	Yes	1.05	0.30	0.92
		Chimp	Yes	1.09	0.57	0.76
	*Au. sediba – H. habilis*	Human	Yes	0.65	0.50	0.12
		Chimp	Yes	0.88	0.55	0.66
	*Au. sediba – H. erectus*	Human	Yes	1.05	0.58	0.85
		Chimp	Yes	1.10	0.82	0.56
**Cranial Analysis 3**	All groups	Human	Yes	1.37	0.87	0.22
**Midface**		Chimp	Yes	1.05	0.65	0.91
	*Au. africanus* – SA early *Homo*	Human	Yes	1.54	0.58	0.45
		Chimp	Yes	0.03	0.00	0.44
	*Au. africanus* – *H. habilis*	Human	Yes	0.97	0.19	0.98
		Chimp	Yes	0.95	0.47	0.93
	*Au. africanus* – *H. erectus*	Human	Yes	2.05	0.78	0.13
		Chimp	Yes	0.99	0.70	0.97
	*Au. africanus – Au. sediba*	Human	Yes	1.33	0.90	0.22
		Chimp	Yes	1.29	0.44	0.71
	*Au. sediba –* SA early *Homo*	Human	No	1.89	0.89	**0.05**
		Chimp	Yes	1.14	0.55	0.80
	*Au. sediba – H. habilis*	Human	Yes	0.06	0.00	0.30
		Chimp	Yes	0.49	0.19	0.37
	*Au. sediba – H. erectus*	Human	Yes	1.62	0.59	0.41
		Chimp	Yes	1.44	0.82	0.26
**Cranial Analysis 4**	All groups	Human	Yes	2.33	0.66	0.19
**Maxilla/Temporal**		Chimp	Yes	1.80	0.74	0.21
	*Au. africanus* – SA early *Homo*	Human	Yes	0.04	0.00	0.51
		Chimp	Yes	2.00	0.31	0.54
	*Au. africanus* – *H. habilis*	Human	Yes	2.20	0.71	0.16
		Chimp	Yes	1.91	0.45	0.43
	*Au. africanus – H. erectus*	Human	Yes	1.99	0.29	0.56
		Chimp	Yes	1.37	0.45	0.65
	*Au. africanus – Au. sediba*	Human	No	3.83	0.78	**0.05**
		Chimp	No	2.18	0.97	**0.00**
	*Au. sediba* – SA early Homo	Human	Yes	2.45	0.56	0.25
		Chimp	Yes	1.87	0.66	0.26
	*Au. sediba – H. habilis*	Human	Yes	2.10	0.56	0.30
		Chimp	Yes	2.25	0.55	0.28
	*Au. sediba – H. erectus*	Human	Maybe	2.94	0.75	**0.08**
		Chimp	Yes	1.56	0.47	0.54
**Cranial Analysis 5**	All groups	Human	Maybe	1.79	0.86	**0.09**
**Neurocranium**		Chimp	No	1.69	0.96	**0.02**
	*Au. africanus – H. erectus*	Human	Yes	2.58	0.44	0.34
		Chimp	Yes	2.06	0.66	0.22
	*Au. africanus – Au. sediba*	Human	Yes	1.82	0.60	0.33
		Chimp	Yes	1.70	0.86	0.11
	*Au. sediba – H. erectus*	Human	Maybe	1.99	0.87	**0.06**
		Chimp	No	1.71	0.97	**0.01**
**Mandibular**	All groups	Human	Yes	0.53	0.51	0.15
**Analysis 1**		Chimp	Yes	0.56	0.36	0.31
	*Au. africanus* – SA early *Homo*	Human	Yes	1.25	0.49	0.72
		Chimp	Yes	0.83	0.09	0.90
	*Au. africanus – H. erectus*	Human	Yes	0.15	0.01	0.40
		Chimp	Yes	0.77	0.46	0.60
	*Au. africanus – Au. sediba* (MH2)	Human	Yes	0.84	0.09	0.91
		Chimp	Yes	1.71	0.68	0.29
	*Au. sediba* (MH2) – SA early *Homo*	Human	Yes	0.42	0.24	0.19
		Chimp	Yes	0.07	0.00	0.32
	*Au. sediba* (MH2) – *H. erectus*	Human	No	0.01	0.00	**0.00**
		Chimp	Yes	−0.57	0.09	0.16
**Mandibular**	All groups	Human	Yes	0.96	0.83	0.87
**Analysis 2**		Chimp	Yes	0.95	0.72	0.87
	*Au. africanus* – SA early *Homo*	Human	Yes	0.19	0.01	0.46
		Chimp	Yes	0.36	0.10	0.30
	*Au. africanus – H. habilis*	Human	Yes	0.74	0.43	0.58
		Chimp	Yes	0.75	0.12	0.82
	*Au. africanus – H. rudolfensis*	Human	Yes	0.98	0.56	0.96
		Chimp	Yes	0.55	0.22	0.43
	*Au. africanus – H. erectus*	Human	Yes	0.96	0.45	0.95
		Chimp	Yes	0.57	0.13	0.59
	*Au. africanus – Au. sediba* (MH1)	Human	Yes	2.06	0.74	0.16
		Chimp	Yes	1.64	0.87	0.11
	*Au. sediba* (MH1) – SA early *Homo*	Human	Yes	1.20	0.43	0.79
		Chimp	Yes	1.09	0.53	0.87
	*Au. sediba* (MH1) – *H. habilis*	Human	Yes	0.00	0.00	0.30
		Chimp	Yes	0.92	0.46	0.88
	*Au. sediba* (MH1) – *H. rudolfensis*	Human	Yes	1.36	0.85	0.27
		Chimp	Yes	1.44	0.36	0.67
	*Au. sediba* (MH1) – *H. erectus*	Human	Yes	1.29	0.77	0.45
		Chimp	Yes	1.32	0.81	0.38
**Mandibular**	All groups	Human	Yes	0.77	0.77	0.34
**Analysis 3**		Chimp	Yes	1.04	0.82	0.89
	*Au. africanus* - SA early *Homo*	Human	Yes	0.38	0.10	0.33
		Chimp	Yes	0.73	0.14	0.78
	*Au. africanus – H. habilis*	Human	Yes	0.76	0.15	0.81
		Chimp	Yes	0.50	0.09	0.58
	*Au. africanus - H. rudolfensis*	Human	Yes	0.16	0.01	0.32
		Chimp	Yes	0.41	0.09	0.43
	*Au. africanus – H. erectus*	Human	Maybe	−0.76	0.20	**0.08**
		Chimp	Yes	0.07	0.00	0.21
	*Au. africanus – Au. sediba* (MH1)	Human	Yes	1.04	0.61	0.92
		Chimp	Yes	0.86	0.31	0.83
	*Au. sediba* (MH1) – SA early *Homo*	Human	Yes	0.65	0.34	0.49
		Chimp	Yes	1.96	0.58	0.31
	*Au. sediba* (MH1) – *H. habilis*	Human	Yes	0.81	0.22	0.82
		Chimp	Yes	1.55	0.74	0.30
	*Au. sediba* (MH1) – *H. rudolfensis*	Human	Yes	1.27	0.69	0.56
		Chimp	Yes	0.92	0.65	0.82
	*Au. sediba* (MH1) – *H. erectus*	Human	Yes	1.15	0.23	0.89
		Chimp	Yes	−0.57	0.04	0.33
**Mandibular**	All groups	Human	Yes	0.76	0.88	0.16
**Analysis 4**		Chimp	Yes	0.80	0.87	0.26
	*Au. africanus* – SA early *Homo*	Human	Yes	1.25	0.33	0.79
		Chimp	Yes	0.85	0.91	0.34
	*Au. africanus – H. habilis*	Human	Yes	0.63	0.38	0.41
		Chimp	Yes	0.87	0.61	0.74
	*Au. africanus – H. rudolfensis*	Human	Yes	1.71	0.41	0.53
		Chimp	Yes	0.12	0.02	0.11
	*Au. africanus – H. erectus*	Human	Maybe	−0.53	0.14	**0.08**
		Chimp	Yes	0.24	0.01	0.52
	*Au. africanus – Au. sediba* (MH2)	Human	Yes	−0.40	0.02	0.33
		Chimp	Maybe	−0.43	0.11	**0.07**
	*Au. sediba* (MH2) – SA early *Homo*	Human	Yes	1.40	0.71	0.42
		Chimp	Yes	1.02	0.33	0.98
	*Au. sediba* (MH2) – *H. habilis*	Human	Yes	0.73	0.33	0.63
		Chimp	Yes	0.97	0.56	0.96
	*Au. sediba* (MH2) – *H. rudolfensis*	Human	Yes	0.64	0.22	0.58
		Chimp	Yes	0.24	0.03	0.31
	*Au. sediba* (MH2) – *H. erectus*	Human	Yes	−0.12	0.01	0.13
		Chimp	Yes	0.39	0.18	0.22
**Mandibular**	All groups	Human	No	0.62	0.74	**0.04**
**Analysis 5**		Chimp	Maybe	0.37	0.18	**0.10**
	*Au. africanus* – SA early *Homo*	Human	Yes	0.13	0.01	0.21
		Chimp	Yes	0.26	0.04	0.21
	*Au. africanus – H. erectus*	Human	Yes	1.10	0.50	0.83
		Chimp	Yes	0.64	0.45	0.26
	*Au. africanus – Au. sediba*	Human	Yes	1.39	0.65	0.39
		Chimp	Yes	1.46	0.57	0.41
	*Au. africanus – Au. sediba* (MH2)	Human	Yes	0.89	0.71	0.64
		Chimp	Yes	1.32	0.58	0.51
	*Au. africanus* – *Au. sediba* (MH1)	Human	Yes	1.33	0.75	0.33
		Chimp	Maybe	1.52	0.84	**0.10**
	*Au. sediba* – SA early *Homo*	Human	No	0.19	0.05	**0.05**
		Chimp	Maybe	0.08	0.01	**0.09**
	*Au. sediba – H. erectus*	Human	Yes	0.14	0.01	0.25
		Chimp	Maybe	−0.11	0.01	**0.07**
	*Au. sediba* (MH2) – SA early *Homo*	Human	Maybe	0.08	0.01	**0.07**
		Chimp	Yes	0.30	0.09	0.11
	*Au. sediba* (MH2) – *H. erectus*	Human	Yes	0.12	0.01	0.12
		Chimp	Yes	0.63	0.10	0.64
	*Au. sediba* (MH1) – SA early *Homo*	Human	Yes	0.35	0.11	0.16
		Chimp	Maybe	0.32	0.14	**0.09**
	*Au. sediba* (MH1) – *H. erectus*	Human	Yes	0.75	0.10	0.80
		Chimp	Yes	−0.41	0.04	0.14

Significant (≤0.05) and near-significant (0.05≤p≤0.10) p-values are shown in bold. Significant values indicate detectable selection (i.e. rejection of drift). Although near-significant values are technically consistent with drift, we indicate ‘Maybe’ here given that test design makes it difficult to reject drift (see Methods).

In the remaining five analyses, all of which focus on mandibular variables, drift is definitively rejected in two instances. In the first mandibular analysis, drift is rejected for the comparison among all non-*Au. africanus* taxa under a human model of variation; this effect appears to be explained by the rejection of drift in the comparison between *Au. sediba* and *H. erectus*, again under a human model, but not *Au. sediba* and the other *Homo* groups. In the final analysis, comparisons between all taxa show significance under a human model. In the remaining comparisons, only the *Au. sediba* comparison with South African *Homo* rejects drift outright, though a number show borderline significance. Unlike the other mandibular analyses, here both the adult and juvenile mandibles are included due to shared morphology, and the analyses are done with them pooled, and individually; although the magnitude of the p-values differs, the overall pattern (i.e. high versus low) is similar. Unlike in the cranial analyses, both analyses of the mandible rejecting drift present slopes less than 1.0, indicating that the first few PCs have minimal between-fossil variation, while less variable components have more than expected.

Results of the rate tests (Table S1) show that deviations from the neutral model occur mainly in the form of directional selection, acting to rapidly differentiate these populations. Interestingly, however, stabilizing selection is detected in two cranial analyses describing the change from *Au. sediba* to *H. habilis*, indicating that selection may have been acting to constrain certain traits in the face between these taxa. These results indicate that there is very little difference between the distributions simulated using the different P matrices for any given heritability. In general, the departures from the neutral model identified in these tests are consistent with results of the slope test for genetic drift.

For the analyses in which a deviation from the neutral model was detected, we reconstruct the magnitude and pattern of selection required to produce: 1) *Au. sediba* from *Au. africanus*, and 2) *Homo* (various species) from *Au. sediba*. We assume that early members of the genus *Homo* are more derived than gracile australopiths (e.g. *Au. sediba* and *Au. africanus*), which is consistent with our understanding of phylogenetic directionality among these groups. We also assume, given the temporal position of *Au. sediba,* as well as its affinities with both *Au. africanus* and *Homo*, that *Au. sediba* is more derived than the *A. africanus* and possibly ancestral to *Homo*. Mean vectors are calculated for each taxon in all analyses where drift is rejected. Difference vectors are calculated as the difference between the mean vectors and are used in combination with estimates of covariance among traits to reconstruct differential selection vectors (Table 3). As an accompanying analysis, we also investigate the bias imparted by sampling error in our estimation of model covariance by reconstructing selection vectors using noise corrected covariance matrices [33] (Text S2). These adjusted selection vectors are shown to be comparable in pattern and magnitude (Table S1). For each of the five comparisons the selection vectors are generally similar in pattern regardless of whether a human or chimpanzee model of V/CV is used (Table 3). Each analysis focuses on a different subset of cranial/mandibular variables, and will be discussed in turn.

**Table 3:** Reconstructed differential selection vectors describing the selection needed to produce *Au. sediba* from *Au. africanus* and later *Homo* from *Au. sediba*.

**Cranial Analysis 3**		**NA-ANS**	**ANS-PRO**	**PRO-NA**	**OR-PRO**	**OR-ZMI**	**ZMI-ANS**		
**Midface**	Difference vector	5.52	9.26	15.81	11.33	14.47	10.10		
*Au. sediba* (MH1) ->	*β*_h_	0.11	0.61	−0.14	0.28	*1.04*	−0.09		
SA early Homo	*β*_c_	**−1.03**	−0.61	*1.04*	−0.68	*1.91*	0.82		
**Cranial Analysis 4**		**ALR-ALR**	**ZMI-ANS**	**ALV-ZMI**	**ALR-ANS**	**ALV-MT**	**POR-MFL**		
**Maxilla/Temporal**	Difference vector	−3.23	−10.71	−12.44	−0.97	−21.08	−4.09		
*Au. africanus* ->	*β*_h_	0.66	0.27	**−1.07**	0.57	**−2.44**	−0.47		
*Au. sediba* (MH1)	*β*_c_	0.86	−0.32	−0.33	−0.58	**−3.23**	−0.03		
**Cranial Analysis 5**		**POR-MFL**	**BR-SON**	**PN-BR**	**AP-BR**	**AP-PN**	**AP-POR**		
**Neurocranium**	Difference vector	3.17	30.23	23.70	19.75	9.44	8.63		
*Au. sediba* (MH1) ->	*β*_h_	0.61	0.68	0.43	−0.04	−0.29	0.15		
*H. erectus*	*β*_c_	0.95	0.85	*1.63*	−0.04	−0.55	−0.02		
**Mandibular Analysis 1**		**INFR-MEN**	**AJUNC-GON**	**AJUNC-MMN**	**MMN-GON**	**ALV-MEN**	**IBB-MEN**		
*Au. sediba* (MH2) ->	Difference vector	2.19	3.35	−1.76	−5.91	−2.88	−0.48		
*H. erectus*	*β*_h_	*1.06*	0.86	−0.53	−0.38	**−1.79**	−0.30		
	*β*_c_	0.28	0.68	−0.06	−0.72	**−1.31**	0.39		
**Mandibular Analysis 5**		**MFO-GON**	**AJUNC-GON**	**AJUNC-MMN**	**M2D-M2M**	**M2M-M1M**	**MMN-MFO**	**AJUNC-MFO**	**IBB-MEN**
*Au. sediba* (combined)	Difference vector	−4.48	0.41	−3.13	−0.59	−0.85	−7.17	−5.15	3.28
-> SA early *Homo*	*β*_h_	−0.99	*1.67*	*1.85*	**−3.13**	0.98	**−4.16**	**−3.71**	*3.22*
	*β*_c_	**−2.41**	*2.96*	*3.89*	−0.18	**−1.90**	**−5.32**	**−6.31**	***2.22***

Significantly negative (values <-1) and significantly positive (values>1) selection are shown in bold and italics respectively. For each comparison, the difference vector (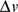) between the two groups is given, as well as the selection vector required to produce that difference, based on human (*β*_h_) and chimpanzee (*β*_c_) V/CV matrices.

**Figure 1:**
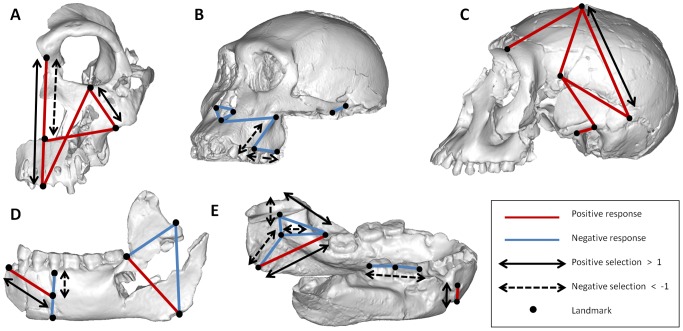
A visual representation of the selection vectors necessary to produce observed differences in cranial and mandibular morphology, as well as the directionality of the observed differences. (A) Cranial analysis 3. Selection required to produce South African early *Homo* (SK 847) from *Au. sediba* is positive for facial length/height and negative for nasal projection, and produces an overall positive morphological response (increase in size). (B) Cranial analysis 4. Selection required to produce *Au. sediba* from *Au. africanus* is negative for the posterior maxilla, and produces a negative response overall (reduction in size). (C) Cranial analysis 5. Selection required to produce *H. erectus* from *Au. sediba* is strongly positive for cranial vault height, and produces a positive response across targeted as well as correlated variables. (D) Mandibular analysis 1. Selection required to produce *H. erectus* from *Au. sediba* is mixed and affects mandibular depth and length, with a consistent morphological response. (E) Mandibular analysis 5. Selection required to produce South African early *Homo* from *Au. sediba* is generally positive for increased height, and negative for width, with the direction of morphological change mapping largely to these selection pressures. Landmark definitions can be found in Table 1. Table 3 presents the selection vectors. Images shown are based on data from both human and chimp V/CV models. Positive and negative morphological change/trait responses are depicted in red and blue respectively. Strongly positive (values>1) and strongly negative (values <−1) selection affecting specific traits are indicated on the images as solid and dashed arrows respectively.

The selection required to produce a South African *Homo* face from an *Au. sediba* face (cranial analysis 3) is strongly to moderately positive for two variables that measure facial length/height (PRO-NA, OR-ZMI), moderately negative for a variable measuring nasal projection (NA-ANS), and weakly negative or positive to nil for the remaining three traits (Figure 1(A)). The actual response to this selective pressure is positive and seen across all variables (signifying overall size increase), indicating that many of the variables are displaying a correlated response to the selective pressure on just two variables, while at least one aspect of morphology (nasal prognathism) is evolving in a direction opposite to the direct force of selection. In the second analysis (cranial analysis 4), which is the only statistically significant analysis directly addressing the *Au. africanus – Au. sediba* transition, the selection required to produce *Au. sediba* maxillary/temporal morphology from *Au. africanus* is strongly to moderately negative for two variables (ALV-MT, ALV-ZMI) and weakly negative or positive for the others (Figure 1(B)). The response to this selective pressure is a reduction in size across all variables; this is most pronounced for the variable that was the direct target of strongly negative selection. However, because ALV-MT is a measure of posterior alveolus length, this reduction may be the result of a lack of complete eruption of the dental row in the juvenile *Au. sediba* [30]. In cranial analysis 5, the selection required to produce the changes in the neurocranium seen between *Au. sediba* and *H. erectus* is moderately positive for a variable measuring cranial vault height (PN-BR) and weakly negative or positive in the remainder (Figure 1(C)). The direction of morphological change is once again positive across all variables, indicating that selection for a taller cranial vault is sufficient to drive the increases seen across the other correlated variables. In mandibular analysis 1, the selection required to produce a *H. erectus* mandible from an adult *Au. sediba* is moderately to weakly positive in two variables (AJUNC-GON, INFR-MEN), moderately negative in one other (ALV-MEN), and weakly negative or positive in the rest (Figure 1(D)), suggesting selective pressure on the depth and length of the body of the mandible or possibly the position of the mental foramen (the location of MEN). The morphological change that results as an action of these selective pressures is consistent with the direction of selection across all of the variables, being a mixture of positive and negative change. For the final analysis (mandibular analysis 5), the selection required to produce a South African *Homo* mandible from an *Au. sediba* one is strongly to moderately positive for three variables (AJUNC-GON, AJUNC-MMN, IBB-MEN) indicating selection for increased height, and especially ramus height. The selection pressure is strongly to moderately negative for three variables (MFO-GON, MMN-MFO, AJUNC-MFO) that measure ramus width (robusticity), and a mixture of strength and signs for the remainder (Figure 1(E)). The direction of morphological change maps directly to the selection pressures, resulting in increased ramus height and decreased ramus width and tooth row. The one exception to this is for the variable AJUNC-MMN, which decreases despite positive selection.

## Discussion

Although natural selection has traditionally been given primacy as the driver of morphological change, strong challenges have been mounted against the adaptationist programme in the past few decades [14], [34], [35]. The methodological and theoretical developments of a host of disciplines applied to a myriad of organisms have made it increasingly clear that drift and gene flow – in addition to selection – have played an often important role in shaping the fates of lineages over evolutionary time [12], [13], [24], [29], [30], [36]–[39]. Yet explanations for phenotypic variation in human evolution remain largely adaptive, regardless of whether this assumption has been tested, and the rise and fall of hominin species is almost exclusively considered a result of how well they are adapted to the environment [40], [41]. The results of the analyses presented here support the view that genetic drift may have played an important role in shaping cranial diversity in hominin evolution. The only previous study to consider this question in early hominins [12] focused on a small set of facial variables. Here, we show that for the entire skull, including the face, neurocranium, and mandible, the diversity seen from ∼2.5–1.5 Ma, as late gracile australopiths evolved into *Homo*, is largely consistent with random evolutionary processes. Given the nature of these tests it is possible that selection was more widespread than detected here, but if so its effects cannot be distinguished from random changes caused by drift. Future research on hominin diversity needs to incorporate non-adaptive process into models of evolutionary change. It will be particularly important to further explore the possible drivers of diversification and innovation within early *Homo*, especially since the presence of multiple closely related lineages [1], [4], [5], [19], [42]–[46] raises the possibility that other factors (e.g. hybridization) may have provided a means for the production of new variation. Finally, it is important to note here that the tests for drift/selection tell us whether the diversity we see is consistent with drift, regardless of the temporal/phylogenetic relationships of these taxa. Therefore, different phylogenetic scenarios linking the taxa described here would not invalidate these results. For example, if (hypothetically) *Au. sediba* were actually ancestral to *Au. africanus* the tests would still reject drift. Moreover, the reconstructed pattern of selection would remain the same, just in reverse (e.g. acting to make small things large and vice versa). Similarly, if the two share a common ancestor that is unsampled in the fossil record, although this ancestor is impossible to model, the conclusions drawn from the tests used here would remain the same.

With respect to natural selection, our results indicate that it provides a necessary explanation for some of the morphological differences seen among *Au. sediba*, *Au. africanus* and *Homo.* This selection, largely directional in nature, generally acts in a manner that is consistent with our current understanding of hominin craniofacial diversity at this time, supporting the notion that an *Au. africanus—Au. sediba—Homo* transition is plausible. For example, in a transition from *Au. africanus* to *Au. sediba*, the negative selection acting on the posterior maxilla may be correlated with the overall trend towards dental reduction as we transition from australopiths to a more *Homo*-like pattern. Similarly, the positive selection acting on the neurocranium that is necessary to produce *Homo* from *Au. sediba* may be correlated with increasing endocranial volume tied to increasing brain size and associated shape changes. In contrast, while most of the cranial changes seen here are greater than expected under a model of drift, those characterizing mandibular change from *Au. sediba* to *Homo* are less than expected. If stabilizing selection was acting to constrain change in the mandible during this transition, this might offer one explanation for the observation of shared aspects of mandibular and dental morphology [20], [47] in these taxa. However, despite the fact that this adaptive transition is plausible, adaptive change is not necessary to transition from a late australopith to early *Homo*, as shown here in the analyses that excluded *Au. sediba*. This may indicate that an evolutionary path to *Homo* without *Au. sediba* is the simpler path. Given current recognition of multiple lineages within early *Homo* [1]–[5], it is also possible that both paths were involved in the production of early *Homo* diversity, and the correlated transition to tool use. This implies that hominins on an australopith—*Homo* path may have marshalled cultural resources to buffer environmental change to a greater extent than those on an australopith—*Au. sediba*—*Homo* path, for whom biological change was an imperative.

## Supporting Information

Figure S1 Regression plots (logged between group vs. logged within group variance) for comparisons in which drift was rejected. (A) Cranial analysis 3. Regression analysis for the comparison of *Au. sediba* (UW88-50; juvenile) and South African early *Homo* using a human model of variance produces an estimated slope of 1.89 with an R^2^ value of 0.89. (B) Cranial analysis 4. Regression analysis for the comparison of *Au. sediba* (UW88-50; juvenile) and *Au. africanus* using a human model of variance produces an estimated slope of 3.83 and R^2^ of 0.79. (C) Cranial analysis 4. Regression analysis for the comparison of *Au. sediba* (UW88-50; juvenile) and *Au. africanus* using a chimpanzee model of variance produces an estimated slope of 2.18 and R^2^ of 0.97. (D) Cranial analysis 5. Regression analysis for the comparison of *Au. sediba* (UW88-50; juvenile) and *H. erectus* produces an estimated slope of 1.71 and R^2^ of 0.97. (E) Mandibular analysis 1. Regression analysis for the comparison of *Au. sediba* (UW88-54; adult) and *H. erectus* produces an estimated slope of 0.01 with a very small R^2^ of 0.001. (F) Mandibular analysis 5. Regression analysis for the comparison of *Au. sediba* and South African early *Homo* produces an estimated slope of 0.19 and R^2^ of 0.05.
(TIF)

Table S1 Results of Lande's generalized genetic distance approach for testing null hypotheses of rates of evolution.
(DOCX)

Table S2 Reconstructed differential selection vectors describing the selection needed to produce *Au. sediba* from *Au. africanus* and later *Homo* from *Au. sediba* using corrected covariance matrices.
(DOCX)

Text S1 Samples.
(DOCX)

Text S2 Covariance matrix correction for differential selection gradient (*β*) reconstruction.
(DOCX)

## References

[1] Antón SC, Potts R, Aiello LC (2014) Evolution of early *Homo*: An integrated biological perspective. Science 345(6192):1236828. 24994657 10.1126/science.1236828

[2] Antón SC (2012) Early *Homo*: Who, when, and where. Curr Anthropol 53(S6):S278–S298.

[3] Kimbel WH (2009) The origin of *Homo* In: Grine FE, Fleagle JG, Leakey REeditors. The First Humans–origin and early evolution of the genus *Homo*. Netherlands: Springer. pp. 31–37.

[4] Leakey MG, Spoor F, Dean MC, Feibel CS, Antón SC, et al (2012) New fossils from Koobi Fora in northern Kenya confirm taxonomic diversity in early *Homo* . Nature 488(7410):201–204. 22874966 10.1038/nature11322

[5] Spoor F, Leakey MG, Gathago PN, Brown FH, Antón SC, et al (2007) Implications of new early *Homo* fossils from Ileret, east of Lake Turkana, Kenya. Nature 448(7154):688–691. 17687323 10.1038/nature05986

[6] Bobe R, Behrensmeyer AK (2004) The expansion of grassland ecosystems in Africa in relation to mammalian evolution and the origin of the genus *Homo* . Palaeogeogr Palaeoclimatol Palaeoecol 207(3):399–420.

[7] Cerling TE (1992) Development of grasslands and savannas in East Africa during the Neogene. Palaeogeogr Palaeoclimatol Palaeoecol 97:241–247.

[8] deMenocal PB, Bloemendal J (1995) Plio-Pleistocene climatic variability in subtropical Africa and the paleoenvironment of hominid evolution. In: Vrba ES, Denton GH, Partridge TC, Burckle LHeditors. Paleoclimate and Evolution, with Emphasis on Human Origins. New Haven: Yale University Press. pp.262–288.

[9] Shackleton N (1995) New data on the evolution of Pliocene climatic variability. In: Vrba ES, Denton GH, Partridge TC, Burckle LHeditors. Paleoclimate and Evolution, with Emphasis on Human Origins. New Haven: Yale University Press. pp.242–248.

[10] Vrba ES (2007) Role of Environmental Stimuli in Hominid Origins. In: Henke W, Rothe H, Tattersall Ieditors. Handbook of Paleoanthropology: Phylogeny of Hominines. Berlin, Heidelberg: Springer. pp.1441–1481.

[11] Wynn JG (2004) Influence of Plio-Pleistocene aridification on human evolution: Evidence from paleosols of the Turkana Basin, Kenya. Am J Phys Anthropol 123(2):106–118. 14730645 10.1002/ajpa.10317

[12] Ackermann RR, Cheverud JM (2004) Detecting genetic drift versus selection in human evolution. Proc Natl Acad Sci USA 101(52):17946–17951. 15604148 10.1073/pnas.0405919102 PMC539739

[13] Weaver TD, Roseman CC, Stringer CB (2007) Were neandertal and modern human cranial differences produced by natural selection or genetic drift? J Hum Evol 53(2):135–145. 17512036 10.1016/j.jhevol.2007.03.001

[14] Gould SJ, Lewontin RC (1979) The spandrels of San Marco and the Panglossian paradigm: A critique of the adaptationist programme. Proc R Soc B 205:581–598. 42062 10.1098/rspb.1979.0086

[15] Kimura M (1968) Evolutionary rate at the molecular level. Nature 217(5129):624–626. 5637732 10.1038/217624a0

[16] Kimura M (1984) The neutral theory of molecular evolution. New York: Cambridge University Press.

[17] Mayr E (1983) How to carry out the adaptationist program? Am Nat 121:324–334.

[18] Pigliucci M, Kaplan J (2000) The fall and rise of Dr Pangloss: adaptationism and the Spandrels paper 20 years later. Trends Ecol Evol 15:66–70. 10652558 10.1016/s0169-5347(99)01762-0

[19] Berger LR, de Ruiter DJ, Churchill SE, Schmid P, Carlson KJ, et al (2010) *Australopithecus sediba*: A new species of *Homo*-like australopith from South Africa. Science 328:195–204. 20378811 10.1126/science.1184944

[20] Irish JD, Guatelli-Steinberg D, Legge SS, de Ruiter DJ, Berger LR (2013) Dental Morphology and the Phylogenetic “Place” of *Australopithecus sediba* . Science 340:1233062. 23580535 10.1126/science.1233062

[21] Lande R (1977) Statistical tests for natural selection on quantitative characters. Evolution 31:442–444. 28563226 10.1111/j.1558-5646.1977.tb01025.x

[22] Lande R (1979) Quantitative genetic analysis of multivariate evolution, applied to brain: body size allometry. Evolution 33:402–416. 28568194 10.1111/j.1558-5646.1979.tb04694.x

[23] Lande R (1980) Genetic variation and phenotypic evolution during allopatric speciation. Am Nat 116:463–479.

[24] Ackermann RR, Cheverud JM (2002) Discerning evolutionary processes in patterns of tamarin (genus *Saguinus*) craniofacial variation. Am J Phys Anthropol 117:260–271. 11842405 10.1002/ajpa.10038

[25] Roseman CC, Willmore KE, Rogers J, Hildebolt C, Sadler BE, et al (2010) Genetic and environmental contributions to variation in baboon cranial morphology. Am J Phys Anthropol 143:p.1–12. 10.1002/ajpa.21341 PMC3258659 20623673

[26] Langergraber KE, Prüfer K, Rowney C, Boesch C, Crockford C, et al (2012) Generation times in wild chimpanzees and gorillas suggest earlier divergence times in great ape and human evolution. Proc Natl Acad Sci 109:15716–15721. 22891323 10.1073/pnas.1211740109 PMC3465451

[27] Yu N, Jensen-Seaman MI, Chemnick L, Ryder O, Li W (2004) Nucleotide diversity in gorillas. Genetics 166:1375–1383. 15082556 10.1534/genetics.166.3.1375 PMC1470796

[28] Lande R, Arnold SJ (1983) The measurement of selection on correlated characters. Evolution 37:1210–1226. 28556011 10.1111/j.1558-5646.1983.tb00236.x

[29] Marroig G, Cheverud JM (2004) Did natural selection or genetic drift produce the cranial diversification of neotropical monkeys? Am Nat 163:417–428. 15026977 10.1086/381693

[30] Marroig G, Vivo M, Cheverud JM (2004) Cranial evolution in sakis (*Pithecia*, Platyrrhini) II: evolutionary processes and morphological integration. J Evol Biol 17:144–155. 15000657 10.1046/j.1420-9101.2003.00653.x

[31] Cheverud JM, Richtsmeier JT (1986) Finite-element scaling applied to sexual dimorphism in rhesus macaque (*Macaca mulatta*) facial growth. Syst Biol 35:381–399.

[32] Ponce de León MS, Zollikofer CP (2001) Neanderthal cranial ontogeny and its implications for late hominid diversity. Nature 412:534–538. 11484052 10.1038/35087573

[33] Marroig G, Melo DA, Garcia G (2012) Modularity, noise, and natural selection. Evolution 66:1506–1524. 22519787 10.1111/j.1558-5646.2011.01555.x

[34] Lewontin RC (1979) Sociobiology as an adaptationist program. Behav Sci 24:5–14. 435219 10.1002/bs.3830240103

[35] Lewontin RC (1991) Biology as ideology: The doctrine of DNA. New York: Harper Collins.

[36] Lofsvold D (1988) Quantitative genetics of morphological differentiation in *Peromyscus*. II. Analysis of selection and drift. Evolution 42:54–67. 28563851 10.1111/j.1558-5646.1988.tb04107.x

[37] Clyde WC, Gingerich PD (1994) Rates of evolution in the dentition of early Eocene *Cantius*: comparison of size and shape. Paleobiology 20(4):506–522.

[38] Lynch M (1990) The rate of morphological evolution in mammals from the standpoint of the neutral expectation. Am Nat 136:727–741.

[39] Roseman CC (2004) Detecting interregionally diversifying natural selection on modern human cranial form by using matched molecular and morphometric data. Proc Natl Acad Sci USA 101:12824–12829. 15326305 10.1073/pnas.0402637101 PMC516480

[40] Klein RG (2009) The human career, human biological and cultural origins, 3rd ed. Chicago, IL: Chicago University Press.

[41] Potts R (2002) Complexity and adaptability in human evolution. In: Goodman M, Moffat ASeditors. Probing human origins. Cambridge, MA: American Academy of Arts and Sciences Cambridge. pp.33–58.

[42] Curnoe D (2010) A review of early *Homo* in southern Africa focusing on cranial, mandibular and dental remains, with the description of a new species (*Homo gautengensis* sp. nov.). Homo 61:151–177. 20466364 10.1016/j.jchb.2010.04.002

[43] Leakey LSB, Tobias PV, Napier J (1964) A new species of the genus *Homo* from Olduvai Gorge. Nature 202:7–9. 14166722 10.1038/202007a0

[44] Bromage TG, Schrenk F, Zonneveld FW (1995) Paleoanthropology of the Malawi Rift: an early hominid mandible from the Chiwondo Beds, northern Malawi. J Hum Evol 28:71–108.

[45] Wood B (1991) Hominid cranial remains, vol. 4 of Koobi Fora research project. Clarendon: Oxford University Press.

[46] Kimbel WH, Walter RC, Johanson DC, Reed KE, Aronson JL, et al (1996) Late Pliocene *Homo* and Oldowan tools from the Hadar Formation (Kada Hadar Member), Ethiopia. J Hum Evol 31:549–561.

[47] de Ruiter DJ, DeWitt TJ, Carlson KB, Brophy JK, Schroeder L, et al (2013) Mandibular remains support taxonomic validity of *Australopithecus sediba* . Science 340:1232997. 23580533 10.1126/science.1232997

[48] Ackermann RR (1998) A Quantitative Assessment of Variability in the Australopithecine, Human, Chimpanzee, and Gorilla Face. Ph.D. Thesis, Washington University, St. Louis.

[49] Harvati K (2003) The Neanderthal taxonomic position: models of intra-and inter-specific craniofacial variation. J Hum Evol 44:107–132. 12604307 10.1016/s0047-2484(02)00208-7

[50] Howells WW (1973) Cranial variation in man: A study by multivariate analysis of patterns of difference among recent human populations. Pap Peabody Mus Archaeol Ethnonel 67:1–259.

[51] Lockwood CA, Kimbel WH, Lynch JM (2004) Morphometrics and hominoid phylogeny: support for a chimpanzee–human clade and differentiation among great ape subspecies. Proc Natl Acad Sci USA 101:4356–4360. 15070722 10.1073/pnas.0306235101 PMC384751

[52] Smith HF (2009) Which cranial regions reflect molecular distances reliably in humans? Evidence from three-dimensional morphology. Am J Hum Biol 21:36–47. 18663742 10.1002/ajhb.20805

[53] von Cramon-Taubadel N (2009) Revisiting the homoiology hypothesis: the impact of phenotypic plasticity on the reconstruction of human population history from craniometric data. J Hum Evol 57:179–190. 19604539 10.1016/j.jhevol.2009.05.009

[54] von Cramon-Taubadel N, Smith HF (2012) The relative congruence of cranial and genetic estimates of hominoid taxon relationships: Implications for the reconstruction of hominin phylogeny. J Hum Evol 62:640–653. 22513382 10.1016/j.jhevol.2012.02.007

[55] Williams FL, Richtsmeier JT (2003) Comparison of mandibular landmarks from computed tomography and 3D digitizer data. Clin Anat 16:494–500. 14566895 10.1002/ca.10095

[56] Willmore KE, Roseman CC, Rogers J, Cheverud JM, Richtsmeier JT (2009) Comparison of mandibular phenotypic and genetic integration between baboon and mouse. Evol Biol 36:19–36. 22212926 10.1007/s11692-009-9056-9 PMC3249057

