# Supplementary figures and images for: Characterizing the Evolutionary Path(s) to Early Homo
Source: PLoS One. 2014 Dec 3;9(12):e114307. doi: 10.1371/journal.pone.0114307 (PMC4255019; doi:10.1371/journal.pone.0114307)

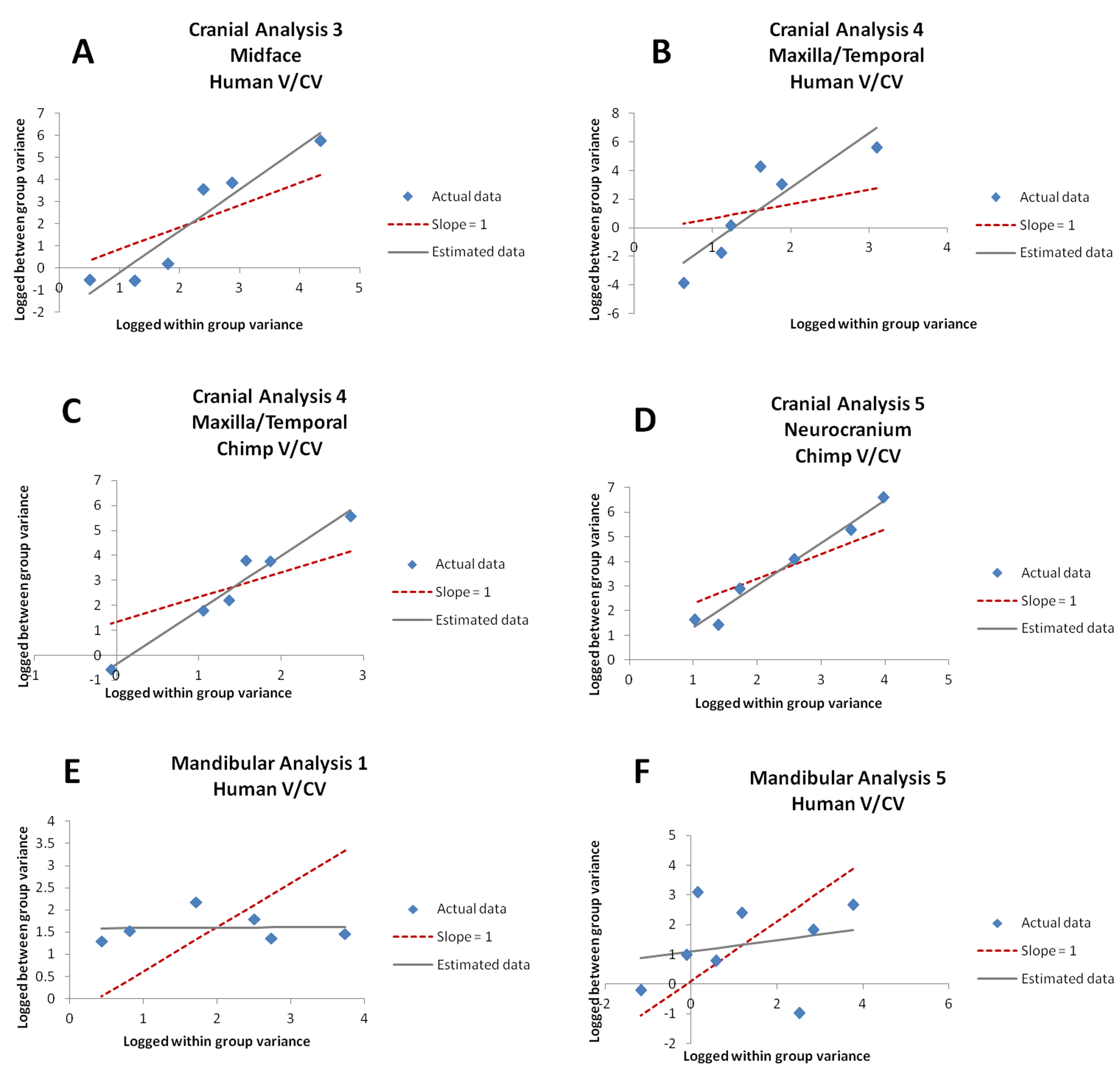

Supplement: Figure S1 — Regression plots (logged between group vs. logged within group variance) for comparisons in which drift was rejected. (A) Cranial analysis 3. Regression analysis for the comparison of Au. sediba (UW88-50; juvenile) and South African early Homo using a human model of variance produces an estimated slope of 1.89 with an R2 value of 0.89. (B) Cranial analysis 4. Regression analysis for the comparison of Au. sediba (UW88-50; juvenile) and Au. africanus using a human model of variance produces an estimated slope of 3.83 and R2 of 0.79. (C) Cranial analysis 4. Regression analysis for the comparison of Au. sediba (UW88-50; juvenile) and Au. africanus using a chimpanzee model of variance produces an estimated slope of 2.18 and R2 of 0.97. (D) Cranial analysis 5. Regression analysis for the comparison of Au. sediba (UW88-50; juvenile) and H. erectus produces an estimated slope of 1.71 and R2 of 0.97. (E) Mandibular analysis 1. Regression analysis for the comparison of Au. sediba (UW88-54; adult) and H. erectus produces an estimated slope of 0.01 with a very small R2 of 0.001. (F) Mandibular analysis 5. Regression analysis for the comparison of Au. sediba and South African early Homo produces an estimated slope of 0.19 and R2 of 0.05. (TIF) [file pone.0114307.s001.tif]
