# Supplementary material for: Characterizing the Evolutionary Path(s) to Early Homo
Source: PLoS One. 2014 Dec 3;9(12):e114307. doi: 10.1371/journal.pone.0114307 (PMC4255019; doi:10.1371/journal.pone.0114307)
Supplement: Table S1 — Results of Lande's generalized genetic distance approach for testing null hypotheses of rates of evolution. (DOCX) [file pone.0114307.s002.docx]

Table S1. Results of Lande’s generalized genetic distance approach for testing null hypotheses of rates of evolution. Results are organized by analysis, species comparison (*Au. sediba* compared to others), heritability assumption, 95% confidence intervals, and point estimates of genetic distances. Point estimates and simulated 95% confidence intervals and median values for the null distribution of Lande’s 1979 generalized genetic distance assume direct ancestor-descendent relationships between the featured group and *Au. sediba*. Simulations take into account both evolutionary and sampling variance. A departure from the neutral model is identified if the genetic distance for each comparison falls outside the range of the confidence interval. If the genetic distance falls above the range then the rate of evolution is fast, indicating directional selection. Similarly, if the genetic distance falls below then the rate of evolution is slow, indicating stabilizing selection.

| **Analysis trait set** | ***Au. sediba* comparisons with other groups** | **Heritability** | **0.025** | **0.5** | **0.975** | **Generalized genetic distance** | **Direction of departure from neutrality** |
| --- | --- | --- | --- | --- | --- | --- | --- |
| **Cranial Analysis 1** | *Au. africanus* | **Human h^2^ = 1.0** | 4.1 | 13.8 | 32.7 | 14 | None |
| **Face** |  | **Human h^2^ = 0.4** | 4.0 | 13.5 | 32.8 | 34 | FAST |
|  |  | **Pan h^2^ = 1.0** | 7.1 | 24.4 | 58.6 | 10 | None |
|  |  | **Pan h^2^ = 0.4** | 4.1 | 13.5 | 32.4 | 25 | None |
|  | *H. habilis* | **Human h^2^ = 1.0** | 7.2 | 24.6 | 57.9 | 5 | SLOW |
|  |  | **Human h^2^ = 0.4** | 7.1 | 24.1 | 57.9 | 12 | None |
|  |  | **Pan h^2^ = 1.0** | 4.0 | 13.8 | 32.5 | 5 | None |
|  |  | **Pan h^2^ = 0.4** | 7.3 | 24.1 | 57.3 | 131 | FAST |
|  | *H. rudolfensis* | **Human h^2^ = 1.0** | 4.0 | 13.7 | 32.5 | 32 | None |
|  |  | **Human h^2^ = 0.4** | 7.0 | 24.1 | 57.3 | 80 | FAST |
|  |  | **Pan h^2^ = 1.0** | 4.2 | 13.8 | 32.4 | 64 | FAST |
|  |  | **Pan h^2^ = 0.4** | 7.4 | 24.2 | 57.9 | 160 | FAST |
|  | South African | **Human h^2^ = 1.0** | 13.5 | 43.7 | 105.8 | 53 | None |
|  | early *Homo* | **Human h^2^ = 0.4** | 19.8 | 66.2 | 158.0 | 132 | None |
|  |  | **Pan h^2^ = 1.0** | 13.4 | 43.8 | 102.5 | 41 | None |
|  |  | **Pan h^2^ = 0.4** | 20.0 | 67.0 | 161.2 | 102 | None |
| **Cranial Analysis 2** | *Au. africanus* | **Human h^2^ = 1.0** | 8.3 | 21.3 | 43.9 | 13 | None |
| **Face** |  | **Human h^2^ = 0.4** | 14.6 | 37.1 | 76.6 | 33 | None |
|  |  | **Pan h^2^ = 1.0** | 8.1 | 21.1 | 43.6 | 20 | None |
|  |  | **Pan h^2^ = 0.4** | 14.1 | 37.3 | 76.7 | 50 | None |
|  | *H. erectus* | **Human h^2^ = 1.0** | 7.9 | 21.0 | 43.1 | 56 | FAST |
|  |  | **Human h^2^ = 0.4** | 14.4 | 37.4 | 76.6 | 140 | FAST |
|  |  | **Pan h^2^ = 1.0** | 8.2 | 21.0 | 42.8 | 53 | FAST |
|  |  | **Pan h^2^ = 0.4** | 14.6 | 37.2 | 76.2 | 133 | FAST |
|  | *H. habilis* | **Human h^2^ = 1.0** | 8.1 | 21.1 | 42.7 | 11 | None |
|  |  | **Human h^2^ = 0.4** | 14.2 | 37.2 | 77.4 | 29 | None |
|  |  | **Pan h^2^ = 1.0** | 8.1 | 21.1 | 43.6 | 8 | SLOW |
|  |  | **Pan h^2^ = 0.4** | 14.4 | 37.3 | 77.5 | 20 | None |
| **Cranial Analysis 3** | *Au. africanus* | **Human h^2^ = 1.0** | 2.2 | 9.8 | 26.4 | 10 | None |
| **Midface** |  | **Human h^2^ = 0.4** | 4.1 | 17.7 | 48.3 | 25 | None |
|  |  | **Pan h^2^ = 1.0** | 2.2 | 10.0 | 27.0 | 11 | None |
|  |  | **Pan h^2^ = 0.4** | 4.1 | 17.6 | 47.7 | 28 | None |
|  | *H. erectus* | **Human h^2^ = 1.0** | 2.3 | 9.9 | 26.4 | 20 | None |
|  |  | **Human h^2^ = 0.4** | 4.2 | 17.9 | 48.3 | 49 | FAST |
|  |  | **Pan h^2^ = 1.0** | 2.3 | 9.8 | 26.8 | 19 | None |
|  |  | **Pan h^2^ = 0.4** | 4.0 | 17.7 | 47.6 | 49 | FAST |
|  | *H. habilis* | **Human h^2^ = 1.0** | 2.4 | 10.0 | 27.0 | 4 | None |
|  |  | **Human h^2^ = 0.4** | 4.1 | 17.5 | 47.8 | 10 | None |
|  |  | **Pan h^2^ = 1.0** | 2.3 | 9.8 | 26.8 | 5 | None |
|  |  | **Pan h^2^ = 0.4** | 4.1 | 17.3 | 47.8 | 13 | None |
|  | South African | **Human h^2^ = 1.0** | 7.5 | 31.7 | 87.2 | 20 | None |
|  | early *Homo* | **Human h^2^ = 0.4** | 11.0 | 47.1 | 127.3 | 51 | None |
|  |  | **Pan h^2^ = 1.0** | 7.5 | 32.4 | 87.2 | 32 | None |
|  |  | **Pan h^2^ = 0.4** | 10.7 | 48.1 | 128.2 | 79 | None |
| **Cranial Analysis 4** | *Au. africanus* | **Human h^2^ = 1.0** | 2.2 | 9.8 | 26.4 | 58 | FAST |
| **Maxilla/Temporal** |  | **Human h^2^ = 0.4** | 4.1 | 17.8 | 48.1 | 146 | FAST |
|  |  | **Pan h^2^ = 1.0** | 2.3 | 9.9 | 27.1 | 70 | FAST |
|  |  | **Pan h^2^ = 0.4** | 4.2 | 17.5 | 47.5 | 175 | FAST |
|  | *H. erectus* | **Human h^2^ = 1.0** | 2.3 | 9.9 | 26.2 | 146 | FAST |
|  |  | **Human h^2^ = 0.4** | 4.0 | 17.8 | 47.7 | 364 | FAST |
|  |  | **Pan h^2^ = 1.0** | 2.3 | 10.0 | 27.2 | 229 | FAST |
|  |  | **Pan h^2^ = 0.4** | 4.2 | 17.7 | 47.1 | 574 | FAST |
|  | *H. habilis* | **Human h^2^ = 1.0** | 2.3 | 9.9 | 27.0 | 30 | FAST |
|  |  | **Human h^2^ = 0.4** | 4.1 | 17.5 | 48.9 | 74 | FAST |
|  |  | **Pan h^2^ = 1.0** | 2.4 | 10.1 | 27.2 | 45 | FAST |
|  |  | **Pan h^2^ = 0.4** | 4.1 | 17.5 | 48.0 | 74 | FAST |
|  | South African | **Human h^2^ = 1.0** | 2.3 | 10.1 | 26.9 | 203 | FAST |
|  | early *Homo* | **Human h^2^ = 0.4** | 3.9 | 17.5 | 47.8 | 509 | FAST |
|  |  | **Pan h^2^ = 1.0** | 2.2 | 10.0 | 27.6 | 266 | FAST |
|  |  | **Pan h^2^ = 0.4** | 4.1 | 17.5 | 46.1 | 665 | FAST |
| **Cranial Analysis 5** | *Au. africanus* | **Human h^2^ = 1.0** | 2.3 | 10.0 | 26.6 | 9 | None |
| **Neurocranium** |  | **Human h^2^ = 0.4** | 4.1 | 17.8 | 46.7 | 23 | None |
|  |  | **Pan h^2^ = 1.0** | 2.3 | 9.9 | 26.8 | 18 | None |
|  |  | **Pan h^2^ = 0.4** | 4.1 | 17.8 | 48.7 | 46 | None |
|  | *H. erectus* | **Human h^2^ = 1.0** | 2.3 | 10.0 | 26.4 | 29 | FAST |
|  |  | **Human h^2^ = 0.4** | 4.2 | 17.7 | 47.8 | 73 | FAST |
|  |  | **Pan h^2^ = 1.0** | 2.3 | 9.9 | 27.0 | 58 | FAST |
|  |  | **Pan h^2^ = 0.4** | 4.0 | 17.5 | 47.3 | 146 | FAST |
| **Mandibular** | *Au. africanus* | **Human h^2^ = 1.0** | 2.3 | 9.8 | 26.6 | 13 | None |
| **Analysis 1** |  | **Human h^2^ = 0.4** | 3.8 | 17.3 | 46.8 | 33 | None |
|  |  | **Pan h^2^ = 1.0** | 2.3 | 10.0 | 26.8 | 9 | None |
|  |  | **Pan h^2^ = 0.4** | 4.0 | 17.6 | 48.2 | 21 | None |
|  | *H. erectus* | **Human h^2^ = 1.0** | 2.3 | 9.9 | 26.8 | 13 | None |
|  |  | **Human h^2^ = 0.4** | 4.1 | 17.5 | 46.9 | 33 | None |
|  |  | **Pan h^2^ = 1.0** | 2.3 | 9.9 | 26.9 | 10 | None |
|  |  | **Pan h^2^ = 0.4** | 4.0 | 17.5 | 47.5 | 26 | None |
|  | South African | **Human h^2^ = 1.0** | 7.6 | 32.4 | 87.9 | 22 | None |
|  | early *Homo* | **Human h^2^ = 0.4** | 11.6 | 48.4 | 132.5 | 56 | None |
|  |  | **Pan h^2^ = 1.0** | 7.3 | 31.7 | 88.2 | 31 | None |
|  |  | **Pan h^2^ = 0.4** | 11.1 | 48.0 | 128.8 | 78 | None |
| **Mandibular** | *Au. africanus* | **Human h^2^ = 1.0** | 2.3 | 10.0 | 26.8 | 8 | None |
| **Analysis 2** |  | **Human h^2^ = 0.4** | 4.0 | 17.6 | 48.2 | 19 | None |
|  |  | **Pan h^2^ = 1.0** | 2.3 | 10.0 | 27.2 | 9 | None |
|  |  | **Pan h^2^ = 0.4** | 4.0 | 17.5 | 47.4 | 22 | None |
|  | *H. erectus* | **Human h^2^ = 1.0** | 2.3 | 9.9 | 26.9 | 31 | FAST |
|  |  | **Human h^2^ = 0.4** | 4.1 | 17.6 | 46.7 | 76 | FAST |
|  |  | **Pan h^2^ = 1.0** | 2.3 | 10.0 | 27.0 | 30 | FAST |
|  |  | **Pan h^2^ = 0.4** | 4.1 | 17.5 | 46.9 | 74 | FAST |
|  | *H. habilis* | **Human h^2^ = 1.0** | 2.3 | 9.9 | 26.5 | 29 | FAST |
|  |  | **Human h^2^ = 0.4** | 3.8 | 17.6 | 47.2 | 72 | FAST |
|  |  | **Pan h^2^ = 1.0** | 2.4 | 10.0 | 26.9 | 16 | None |
|  |  | **Pan h^2^ = 0.4** | 4.1 | 17.5 | 47.9 | 41 | None |
|  | *H. rudolfensis* | **Human h^2^ = 1.0** | 2.2 | 10.0 | 26.8 | 25 | None |
|  |  | **Human h^2^ = 0.4** | 4.0 | 17.6 | 47.0 | 62 | FAST |
|  |  | **Pan h^2^ = 1.0** | 2.2 | 9.9 | 27.0 | 22 | None |
|  |  | **Pan h^2^ = 0.4** | 4.1 | 17.3 | 47.6 | 55 | FAST |
|  | South African | **Human h^2^ = 1.0** | 7.3 | 31.9 | 86.7 | 39 | None |
|  | early *Homo* | **Human h^2^ = 0.4** | 11.4 | 47.9 | 129.7 | 97 | None |
|  |  | **Pan h^2^ = 1.0** | 7.6 | 32.4 | 88.6 | 67 | None |
|  |  | **Pan h^2^ = 0.4** | 10.9 | 48.0 | 132.4 | 169 | FAST |
| **Mandibular** | *Au. africanus* | **Human h^2^ = 1.0** | 2.2 | 10.0 | 27.2 | 10 | None |
| **Analysis 3** |  | **Human h^2^ = 0.4** | 4.1 | 17.5 | 46.4 | 26 | None |
|  |  | **Pan h^2^ = 1.0** | 2.3 | 10.0 | 26.9 | 15 | None |
|  |  | **Pan h^2^ = 0.4** | 4.1 | 17.9 | 47.8 | 37 | None |
|  | *H. erectus* | **Human h^2^ = 1.0** | 2.3 | 10.0 | 27.3 | 30 | FAST |
|  |  | **Human h^2^ = 0.4** | 4.1 | 17.6 | 46.8 | 76 | FAST |
|  |  | **Pan h^2^ = 1.0** | 2.3 | 9.9 | 26.7 | 21 | None |
|  |  | **Pan h^2^ = 0.4** | 4.1 | 17.6 | 47.4 | 54 | FAST |
|  | *H. habilis* | **Human h^2^ = 1.0** | 2.3 | 10.0 | 27.3 | 47 | FAST |
|  |  | **Human h^2^ = 0.4** | 4.1 | 17.6 | 46.9 | 118 | FAST |
|  |  | **Pan h^2^ = 1.0** | 2.3 | 10.0 | 27.1 | 22 | None |
|  |  | **Pan h^2^ = 0.4** | 4.1 | 17.8 | 48.1 | 55 | FAST |
|  | *H. rudolfensis* | **Human h^2^ = 1.0** | 2.2 | 9.9 | 26.8 | 22 | None |
|  |  | **Human h^2^ = 0.4** | 4.1 | 17.3 | 47.6 | 55 | FAST |
|  |  | **Pan h^2^ = 1.0** | 2.2 | 10.1 | 27.2 | 27 | None |
|  |  | **Pan h^2^ = 0.4** | 4.1 | 17.6 | 48.5 | 68 | FAST |
|  | South African | **Human h^2^ = 1.0** | 7.5 | 32.2 | 85.8 | 54 | None |
|  | early *Homo* | **Human h^2^ = 0.4** | 11.4 | 48.2 | 132.3 | 136 | FAST |
|  |  | **Pan h^2^ = 1.0** | 7.7 | 32.2 | 88.1 | 38 | None |
|  |  | **Pan h^2^ = 0.4** | 11.0 | 48.4 | 128.4 | 95 | None |
| **Mandibular** | *Au. africanus* | **Human h^2^ = 1.0** | 2.2 | 10.0 | 26.3 | 15 | None |
| **Analysis 4** |  | **Human h^2^ = 0.4** | 4.2 | 17.6 | 46.8 | 38 | None |
|  |  | **Pan h^2^ = 1.0** | 2.3 | 9.9 | 27.1 | 20 | None |
|  |  | **Pan h^2^ = 0.4** | 4.1 | 17.6 | 46.6 | 49 | FAST |
|  | *H. erectus* | **Human h^2^ = 1.0** | 2.3 | 10.0 | 26.7 | 4 | None |
|  |  | **Human h^2^ = 0.4** | 4.2 | 17.5 | 47.3 | 10 | None |
|  |  | **Pan h^2^ = 1.0** | 2.3 | 10.0 | 26.8 | 3 | None |
|  |  | **Pan h^2^ = 0.4** | 4.0 | 17.6 | 48.1 | 8 | None |
|  | *H. habilis* | **Human h^2^ = 1.0** | 2.3 | 10.1 | 26.7 | 8 | None |
|  |  | **Human h^2^ = 0.4** | 3.9 | 18.0 | 47.6 | 19 | None |
|  |  | **Pan h^2^ = 1.0** | 2.3 | 10.0 | 26.8 | 6 | None |
|  |  | **Pan h^2^ = 0.4** | 4.0 | 17.4 | 47.0 | 14 | None |
|  | *H. rudolfensis* | **Human h^2^ = 1.0** | 2.3 | 9.9 | 26.6 | 21 | None |
|  |  | **Human h^2^ = 0.4** | 4.3 | 17.5 | 46.7 | 53 | FAST |
|  |  | **Pan h^2^ = 1.0** | 2.3 | 9.9 | 26.6 | 17 | None |
|  |  | **Pan h^2^ = 0.4** | 4.1 | 17.4 | 47.3 | 41 | None |
|  | South African | **Human h^2^ = 1.0** | 7.4 | 31.7 | 85.2 | 16 | None |
|  | early *Homo* | **Human h^2^ = 0.4** | 11.4 | 48.0 | 131.6 | 39 | None |
|  |  | **Pan h^2^ = 1.0** | 7.7 | 31.8 | 86.9 | 15 | None |
|  |  | **Pan h^2^ = 0.4** | 11.3 | 47.8 | 128.4 | 36 | None |
| **Mandibular** | *Au. africanus* | **Human h^2^ = 1.0** | 3.1 | 10.2 | 24.2 | 16 | None |
| **Analysis 5** |  | **Human h^2^ = 0.4** | 4.7 | 15.4 | 36.4 | 39 | FAST |
|  |  | **Pan h^2^ = 1.0** | 3.1 | 10.3 | 24.4 | 10 | None |
|  |  | **Pan h^2^ = 0.4** | 4.6 | 15.5 | 37.0 | 24 | None |
|  | *H. erectus* | **Human h^2^ = 1.0** | 2.9 | 10.2 | 24.4 | 27 | FAST |
|  |  | **Human h^2^ = 0.4** | 4.6 | 15.3 | 36.3 | 68 | FAST |
|  |  | **Pan h^2^ = 1.0** | 3.0 | 10.2 | 24.0 | 24 | FAST |
|  |  | **Pan h^2^ = 0.4** | 4.6 | 15.2 | 36.6 | 60 | FAST |
|  | South African | **Human h^2^ = 1.0** | 10.7 | 36.8 | 88.3 | 57 | None |
|  | early *Homo* | **Human h^2^ = 0.4** | 13.5 | 47.8 | 113.5 | 142 | FAST |
|  |  | **Pan h^2^ = 1.0** | 10.8 | 36.8 | 88.8 | 76 | FAST |
|  |  | **Pan h^2^ = 0.4** | 14.1 | 48.1 | 112.8 | 189 | FAST |
