# Supplementary material for: Characterizing the Evolutionary Path(s) to Early Homo
Source: PLoS One. 2014 Dec 3;9(12):e114307. doi: 10.1371/journal.pone.0114307 (PMC4255019; doi:10.1371/journal.pone.0114307)
Supplement: Table S2 — Reconstructed differential selection vectors describing the selection needed to produce Au. sediba from Au. africanus and later Homo from Au. sediba using corrected covariance matrices. (DOCX) [file pone.0114307.s003.docx]

Table S2. Reconstructed differential selection vectors describing the selection needed to produce *Au. sediba* from *Au. africanus* and later *Homo* from *Au. sediba* using corrected covariance matrices. Significantly negative (values <-1) and significantly positive (values >1) selection are shown in bold and italics respectively. For each comparison, the difference vector ($\Delta v$) between the two groups is given, as well as the selection vector required to produce that difference, based on human (*β* _h_) and chimpanzee (*β* _c_) V/CV matrices.

| **Cranial Analysis 3** |  | **NA-ANS** | **ANS-PRO** | **PRO-NA** | **OR-PRO** | **OR-ZMI** | **ZMI-ANS** |  |  |
| --- | --- | --- | --- | --- | --- | --- | --- | --- | --- |
| **Midface** | Difference vector | 5.52 | 9.26 | 15.81 | 11.33 | 14.47 | 10.10 |  |  |
| *Au. sediba* (MH1) -> | *β* _h_ | -0.09 | 0.49 | 0.13 | 0.03 | 0.92 | 0.11 |  |  |
| SA early *Homo* | *β* _c_ | -0.15 | 0.16 | 0.14 | -0.54 | *1.91* | 0.74 |  |  |
| **Cranial Analysis 4** |  | **ALR-ALR** | **ZMI-ANS** | **ALV-ZMI** | **ALR-ANS** | **ALV-MT** | **POR-MFL** |  |  |
| **Maxilla/Temporal** | Difference vector | -3.23 | -10.71 | -12.44 | -0.97 | -21.08 | -4.09 |  |  |
| *Au. africanus* -> | *β* _h_ | 0.64 | 0.25 | **-1.05** | 0.61 | **-2.45** | -0.48 |  |  |
| *Au. sediba* (MH1) | *β* _c_ | 0.49 | -0.31 | -0.38 | 0.20 | **-3.05** | -0.24 |  |  |
| **Cranial Analysis 5** |  | **POR-MFL** | **BR-SON** | **PN-BR** | **AP-BR** | **AP-PN** | **AP-POR** |  |  |
| **Neurocranium** | Difference vector | 3.17 | 30.23 | 23.70 | 19.75 | 9.44 | 8.63 |  |  |
| *Au. sediba* (MH1) -> | *β* _h_ | 0.39 | 0.67 | 0.43 | -0.01 | -0.33 | 0.25 |  |  |
| *H. erectus* | *β* _c_ | 0.60 | 0.84 | *1.60* | -0.02 | -0.52 | 0.04 |  |  |
| **Mandibular Analysis 1** |  | **INFR-MEN** | **AJUNC-GON** | **AJUNC-MMN** | **MMN-GON** | **ALV-MEN** | **IBB-MEN** |  |  |
| *Au. sediba* (MH2) -> | Difference vector | 2.19 | 3.35 | -1.76 | -5.91 | -2.88 | -0.48 |  |  |
| *H. erectus* | *β* _h_ | 0.25 | 0.34 | -0.11 | -0.35 | -0.20 | -0.01 |  |  |
|  | *β* _c_ | 0.20 | 0.34 | -0.10 | -0.44 | -0.24 | -0.03 |  |  |
| **Mandibular Analysis 5** |  | **MFO-GON** | **AJUNC-GON** | **AJUNC-MMN** | **M2D-M2M** | **M2M-M1M** | **MMN-MFO** | **AJUNC-MFO** | **IBB-MEN** |
| *Au. sediba* (combined) | Difference vector | -4.48 | 0.41 | -3.13 | -0.59 | -0.85 | -7.17 | -5.15 | 3.28 |
| -> SA early *Homo* | *β* _h_ | -0.98 | *1.57* | *1.68* | -0.59 | -0.61 | **-3.75** | **-3.39** | *2.92* |
|  | *β* _c_ | **-2.10** | *2.53* | *3.17* | -0.72 | **-1.09** | **-4.45** | **-5.25** | *1.98* |
