# Supplementary material for: Characterizing the Evolutionary Path(s) to Early Homo
Source: PLoS One. 2014 Dec 3;9(12):e114307. doi: 10.1371/journal.pone.0114307 (PMC4255019; doi:10.1371/journal.pone.0114307)
Supplement: Text S1 — Samples. (DOCX) [file pone.0114307.s004.docx]

**Text S1. Samples**

The fossil specimens used in each analysis are as follows:

**Cranial Analysis 1**

*Au. africanus*: Sts 5, Sts 71

*Au. sediba*: MH1

South African early *Homo*: SK 847

*H. habilis*: KNM-ER 1813, OH 24

*H. rudolfensis*: KNM-ER 1470

**Cranial Analysis 2**

*Au. africanus*: Sts 5, Sts 52, Sts 71, Stw 505

*Au. sediba*: MH1

*H. habilis*: KNM-ER 1813, OH 24

*H. erectus*: KNM-WT 15000

**Cranial Analysis 3**

*Au. africanus*: Sts 5, Sts 52, Sts 71, Stw 13, Stw 505

*Au. sediba*: MH1

South African early *Homo*: SK 847

*H. habilis*: KNM-ER 1813, OH 24

*H. erectus*: KNM-ER 3733

**Cranial Analysis 4**

*Au. africanus*: Sts 5, Sts 71

*Au. sediba*: MH1

South African early *Homo*: Stw 53

*H. habilis*: KNM-ER 1813, OH 24

*H. erectus*: KNM-WT 15000

**Cranial Analysis 5**

*Au. africanus*: Stw 505

*Au. sediba*: MH1

*H. erectus*: KNM-ER 3883, KNM-ER 42700, KNM-WT 15000

**Mandibular Analysis 1**

*Au. africanus*: Sts 36

*Au. sediba*: MH2

South African early *Homo*: SK 15

*H. erectus*: KNM-ER 992, KNM-WT 15000

**Mandibular Analysis 2**

*Au. africanus*: MLD 2, Sts 36, Sts 52b, Stw 513

*Au. sediba*: MH1

South African early *Homo*: SK 15

*H. habilis*: KNM-ER 820, OH 13

*H. rudolfensis*: KNM-ER 1482, KNM-ER 1802*

*H. erectus*: KNM-BK 67, KNM-BK 8518, KNM-ER 992, KNM-WT 15000, OH 22

**Mandibular Analysis 3**

*Au. africanus*: MLD 2, MLD 40, Sts 7, Sts 36, Sts 52b, Stw 327, Stw 513

*Au. sediba*: MH1

South African early *Homo*: SK 15, SK 45

*H. habilis*: KNM-ER 820, KNM-ER 1501, OH 13, OH 37

*H. rudolfensis*: KNM-ER 1482, KNM-ER 1801, KNM-ER 1802*

*H. erectus*: KNM-BK 67, KNM-BK 8518, KNM-ER 992, KNM-ER 3734, KNM-WT 15000, OH 22

**Mandibular Analysis 4**

*Au. africanus*: MLD 2, Sts 7, Sts 36, Sts 52b

*Au. sediba*: MH2

South African early *Homo*: SK 15

*H. habilis*: KNM-ER 820, OH 13

*H. rudolfensis*: KNM-ER 1482

*H. erectus*: KNM-BK 67, KNM-BK 8518, KNM-ER 730, KNM-ER 992, KNM-WT 15000, OH 22

**Mandibular Analysis 5**

*Au. africanus*: Sts 36

*Au. sediba*: MH1, MH2

South African early *Homo*: SK 15

*H. erectus*: KNM-WT 15000

All data were collected from original specimens, except Stw 53 where a cast was utilized. South African early *Homo* was analyzed as a separate group. Accession number site abbreviations and repository information for the fossil data are as follows: Sts (Sterkfontein site pre-1968) and SK (Swartkrans site pre-1968) from the Ditsong Museum, Pretoria, South Africa; Stw (Sterkfontein site 1968-present), MLD (Makapansgat limework deposits), and MH (Malapa hominin) from the Evolutionary Studies Institute, University of the Witwatersrand, South Africa; KNM-BK (Kenya National Museums Baringo Kapthurin; KNM-ER (Kenya National Museums east Rudolf), and KNM-WT (Kenya National Museums west Turkana) from the National Museums of Kenya, Nairobi; OH (Olduvai hominin) from the National Museum of Tanzania, Dar es Salaam. Permits for fossil data collection in Kenya and Tanzania were obtained from the National Council for Science and Technology (research permit number NCST/RRI/12/1/BS/217/5), and the Tanzanian Commission for Science and Technology (research permit number 2010-379-CC-2010-185), respectively. No permit was required for South African hominin fossil collections.

Extant cranial and mandibular material consists of samples of adult African *Homo sapiens* (*n* = 100; Raymond Dart Collection, University of the Witwatersrand, ZA and Iziko Museums of South Africa, Cape Town, ZA) comprised of Bantu-speakers and Khoesan individuals, and *Pan troglodytes* (*n* = 80; Hamann-Todd Collection, Cleveland Museum of Natural History, Ohio, USA). Extant samples consist of roughly equal numbers of males and females.

______________

* Mandibular analyses 2 and 3 were also performed without specimen KNM-ER 1802, following its taxonomic reassignment in Anton *et al*. [1]. However, removing KNM-ER 1802 had very little effect on our results, therefore, here, we follow conventional taxonomic affiliations

**References**

1. Antón SC, Potts R, Aiello LC (2014) Evolution of early *Homo*: An integrated biological perspective. Science 345(6192): 1236828.
