# Supplementary material for: Characterizing the Evolutionary Path(s) to Early Homo
Source: PLoS One. 2014 Dec 3;9(12):e114307. doi: 10.1371/journal.pone.0114307 (PMC4255019; doi:10.1371/journal.pone.0114307)
Supplement: Text S2 — Covariance matrix correction for differential selection gradient (β) reconstruction. (DOCX) [file pone.0114307.s005.docx]

**Text S2. Covariance matrix correction for differential selection gradient (***β* **) reconstruction**

**Method.** To investigate and account for the error in estimated covariance matrices and the possible impact this error may have on the calculation of selection gradients (*β* ), we use a matrix correction extension approach developed by Hayden and Twede [1, 2], modified and tested by Marroig *et al*. [3]. Following Marroig *et al*. [3], the difficulty in estimating covariance matrices is a feature of the modularity of biological systems whereby the variation within and between different modules is distributed unequally [3, 4, 5]. The error or noise involved in this estimation is exacerbated by measurement error, as well as small sample sizes, and is particularly significant for analyses requiring the calculation of inverted matrices, such as the reconstruction of differential selection gradients [3]. Matrix inversion, by nature, amplifies the degree of noise in a matrix, and therefore this noise tends to dominate the inverted matrix [3]. The approach in Marroig *et al*. [3] is used to account for this effect in directional selection reconstruction by controlling the noise in matrix estimation. This method is based on the following matrix equation: $C=V^{T}\Lambda V$ where $C$ is the matrix, $V$is a square matrix of normalized eigenvectors, $V^{T}$is a transposed matrix of normalized eigenvectors, and $\Lambda$ is a square diagonal matrix of eigenvalues$\lambda_{i}$. The inverse of $C$ is then given by: $C^{-1}=V^{T}\Lambda^{-1}V$ where $\Lambda^{-1}$ denotes the inverse of $\Lambda$ and the diagonal of $\Lambda^{-1}$ represents the inverse of each eigenvalue$\frac{1}{\lambda_{i}}$ . The smallest eigenvalues retain the most noise and will become the dominant feature of the inverted matrix$C^{-1}$ . In the extension approach, on a case-by-case basis, these trailing eigenvalues are replaced by the last reliable eigenvalue, found by calculating the second derivative of consecutive eigenvalues and evaluating the variance between these derivatives in groups of 2-5 to determine at which point and at what eigenvalue this variance tends to zero, therefore giving us the cut-off point for reliability [3]. A new covariance matrix is then calculated from the corrected$\Lambda^{-1}$, and used in selection gradient reconstruction.

**Results.** Noise corrected covariance matrices were estimated for each extant model for the five analyses producing rejections of genetic drift. Differential selection vectors were then reconstructed from these adjusted matrices. Each eigenvalue reliability cut-off point was determined for each case separately. In general, the pattern of results is consistent between the corrected and uncorrected selection vectors, however there are some discrepancies in magnitude. In cranial analysis 3 the selection required to produce a South African *Homo* face from an *Au. sediba* face is still strongly positive for facial length/facial height (OR-ZMI), however the remaining variables display weaker negative or positive selection gradients compared to their uncorrected vectors counterparts (Table S2). Similarly, in mandibular analysis 1, the selection required to produce a *H. erectus* mandible from an adult *Au. sediba* is weaker for all variables when reconstructed using the corrected covariance matrices, displaying no significantly strong positive or negative selection acting on any traits (Table S2). Nevertheless, the pattern of selection vectors (the sequence of negative and positive selection gradient values) is comparable to the pattern calculated using the uncorrected covariance matrices. This suggests that our original covariance matrices, calculated using sample sizes of between 70 and 100 individuals, are suitable estimates for our model covariances. The disparities described above therefore do not affect our final conclusion, and the uncorrected selection vectors are used for our interpretation.

**References**

1. Twede ER, Hayden AF (2004) Refinement and generalization of the extension method of covariance matrix inversion by regularization for spectral filtering optimization imaging. Proc SPIE 5159: 299-306.

2. Hayden AF, Twede ER (2002) Observations on the relationship between eigenvalues, instrument noise and detection. Proc SPIE 4816: 355-362.

3. Marroig G, Melo DA, Garcia G (2012) Modularity, noise, and natural selection. Evolution 66: 1506-1524.

4. Wagner GP, Pavlicev M, Cheverud JM (2007) The road to modularity. Nat Rev Genet 8: 921-931.

5. Olson EC, Miller RL (1958) Morphological integration. Chicago, IL: University of Chicago Press.
